# Synchrotron microcrystal native-SAD phasing at a low energy

**DOI:** 10.1107/S2052252519004536

**Published:** 2019-05-03

**Authors:** Gongrui Guo, Ping Zhu, Martin R. Fuchs, Wuxian Shi, Babak Andi, Yuan Gao, Wayne A. Hendrickson, Sean McSweeney, Qun Liu

**Affiliations:** a Biology Department, Brookhaven National Laboratory, Upton, NY 11973, USA; b Photon Science, NSLS-II, Brookhaven National Laboratory, Upton, NY 11973, USA; c Department of Biochemistry and Molecular Biophysics, Columbia University, New York, NY 10032, USA; d Department of Physiology and Cellular Biophysics, Columbia University, New York, NY 10032, USA

**Keywords:** S-SAD, native SAD, microcrystals, microdiffraction, radiation damage, multiple crystals, anomalous diffraction, low-energy X-rays

## Abstract

*De novo* structural evaluation of native biomolecules from single-wavelength anomalous diffraction (SAD) is a challenge because of the weakness of the anomalous scattering. The anomalous scattering from relevant native elements – primarily sulfur in proteins and phospho­rus in nucleic acids – increases as the X-ray energy decreases toward their *K*-edge transitions. Thus, measurements at a lowered X-ray energy are promising for making native SAD routine and robust. For microcrystals with sizes less than 10 µm, native-SAD phasing at synchrotron microdiffraction beamlines is even more challenging because of difficulties in sample manipulation, diffraction data collection and data analysis. Native-SAD analysis from microcrystals by using X-ray free-electron lasers has been demonstrated but has required use of thousands of thousands of microcrystals to achieve the necessary accuracy. Here it is shown that by exploitation of anomalous microdiffraction signals obtained at 5 keV, by the use of polyimide wellmounts, and by an iterative crystal and frame-rejection method, microcrystal native-SAD phasing is possible from as few as about 1 200 crystals. Our results show the utility of low-energy native-SAD phasing with microcrystals at synchrotron microdiffraction beamlines.

## Abstract 2

Low-energy native-SAD phasing from microcrystals of less than 10 micrometres in size is demonstrated at a synchrotron microdiffraction beamline.

## 1. Introduction   

Anomalous diffraction can produce the phase information needed to determine crystal structures, and the methods of multi- and single-wavelength anomalous diffraction (MAD and SAD) now predominate for *de novo* structure determination of biological macromolecules (Hendrickson, 2014 ▸). Historically, conventional heavy-atom derivatizations and seleno­methio­nine substitutions in proteins have generated the phasing elements in such analyses; however, even the light elements present in virtually all biomolecules, sulfur (*Z* = 16) in proteins and phospho­rus (*Z* = 15) in nucleic acids, were shown quite early to suffice in favorable cases (Hendrickson & Teeter, 1981 ▸; Dauter *et al.*, 1999 ▸; Liu *et al.*, 2000 ▸; Yang *et al.*, 2003 ▸). Obtaining structures without the need for heavy atoms is appealing but the weakness of anomalous signals from light elements has complicated the generality of the approach. With technical advances such as pixel array detectors (Broennimann *et al.*, 2006 ▸), rational treatment of multiple crystals (Liu *et al.*, 2012 ▸, 2013 ▸; Akey *et al.*, 2014 ▸), multi-axis data collection (Weinert *et al.*, 2015 ▸) and improved structure-determination methods (de La Fortelle & Bricogne, 1997 ▸; Sheldrick, 2010 ▸; Bunkóczi *et al.*, 2015 ▸; Terwilliger *et al.*, 2016 ▸), SAD phasing from native biomolecules (native SAD) is becoming routine and robust (Dauter, 2006 ▸; Doutch *et al.*, 2012 ▸; Garman, 2014 ▸; Liu & Hendrickson, 2015 ▸; Rose *et al.*, 2015 ▸; Rose & Wang, 2016 ▸; Liu & Hendrickson, 2017 ▸).

Successful exploitation of native SAD for micron-sized crystals remains a particular challenge. Because of radiation-damage limitations, many microcrystals are required to obtain a complete set of diffraction data, and complications from merging of multi-crystal data exacerbate the extraction of intrinsically weak anomalous signals from microcrystal diffraction patterns. X-ray free-electron lasers are well suited for microcrystals (Chapman *et al.*, 2011 ▸; Boutet *et al.*, 2012 ▸). These ‘super-brilliant’ beams make ‘diffraction before destruction’ possible and enable serial femtosecond crystallography (SFX) (Chapman *et al.*, 2011 ▸; Boutet *et al.*, 2012 ▸). Successful SFX SAD has been demonstrated from the relatively strong anomalous signals of higher *Z* elements, including Gd (*Z* = 64) (Barends *et al.*, 2014 ▸), Zn (*Z* = 30) (Hunter *et al.*, 2016 ▸) and Hg (*Z* = 80) (Yamashita *et al.*, 2017 ▸). SFX SAD phasing has also been demonstrated for native, low-*Z* elements like sulfur (*Z* = 16) or sulfur plus chlorine (*Z* = 17) in tests with lysozyme (Nakane *et al.*, 2015 ▸), thaumatin (Nass *et al.*, 2016 ▸) and the A_2A_ adenosine receptor (Batyuk *et al.*, 2016 ▸). These native-SAD experiments were conducted at 7 keV, 6 keV and 6 keV, respectively, and they were evaluated from 179 574, 363 000, and 578 620 indexed images (crystals), respectively. Because of the requirement of a large sample quantity and limitations on access, SFX native-SAD phasing is time-consuming, expensive and not accessible to most users.

Synchrotron beamlines optimized for microdiffraction have become available for routine user access (Perrakis *et al.*, 1999 ▸; Flot *et al.*, 2010 ▸; Smith *et al.*, 2012 ▸; Fuchs *et al.*, 2016 ▸; Yamamoto *et al.*, 2017 ▸). Microcrystal synchrotron crystallography is now becoming increasingly attractive at microdiffraction beamlines (Ji *et al.*, 2010 ▸; Zeldin *et al.*, 2013*a* ▸; Gati *et al.*, 2014 ▸; Stellato *et al.*, 2014 ▸; Botha *et al.*, 2015 ▸; Coquelle *et al.*, 2015 ▸; Nogly *et al.*, 2015 ▸; Beyerlein *et al.*, 2017 ▸; Diederichs & Wang, 2017 ▸; Martin-Garcia *et al.*, 2017 ▸; Meents *et al.*, 2017 ▸; Gao *et al.*, 2018 ▸). Using crystals of 10–20 µm in size, synchrotron SAD phasing has been demonstrated with higher *Z* elements including iodine (*Z* = 53) and selenium (*Z* = 34) as well as for low-*Z* native-SAD (Melnikov *et al.*, 2017 ▸; Huang *et al.*, 2018 ▸). With microcrystals of less than 10 µm, the weakness of their diffraction and challenges in sample manipulation and diffraction analysis so far have prevented practical applications at synchrotron sources.

We and others have shown the impact of crystal sizes on native SAD and suggested using low energy for optimizing microcrystal native-SAD analysis (Liu *et al.*, 2014 ▸; Liebschner *et al.*, 2016 ▸; Wagner *et al.*, 2016 ▸; Guo *et al.*, 2018 ▸). With crystals smaller than 10 µm, sample absorption of low-energy X-rays (3–5 keV) can be tolerated so as to permit enhancement of the imaginary component of anomalous diffraction signals (denoted as f") from sulfur. Here, we describe a microcrystal native-SAD experiment at the Frontier Microfocusing Macromolecular Crystallographic Beamline (17ID-2, FMX) at the National Synchrotron Light Source II (NSLS-II). By collecting multi-crystal data at an energy of 5 keV (λ = 2.48 Å) from crystals on low-absorbance polyimide wellmounts, and by using an iterative outlier rejection strategy, we demonstrate that structure determination by synchrotron-based microcrystal native-SAD is feasible from 32 323 diffraction patterns, which were collected from fewer than 1 200 thaumatin microcrystals.

## 2. Methods   

### 2.1. Sample preparation   

Microcrystals of thaumatin were prepared and handled as previously reported (Guo *et al.*, 2018 ▸). Briefly, microcrystals were filtrated three times through an 8 µm Whatman Nuclepore Track-Etched membrane (GE Healthcare). Microcrystals were concentrated by centrifugation, and supernatant was removed. Crystal slurries were loaded to custom-made polyimide micro-sized wellmounts with 2 µm holes for solvent to pass through. Solvents were then removed by touching the bottom side of the mounts using a fine-tip filter paper followed by manually plunging into liquid nitro­gen for rapid freezing. Cryoprotectant was not added prior to freezing.

### 2.2. Microdiffraction data collection   

**Figure 1:**
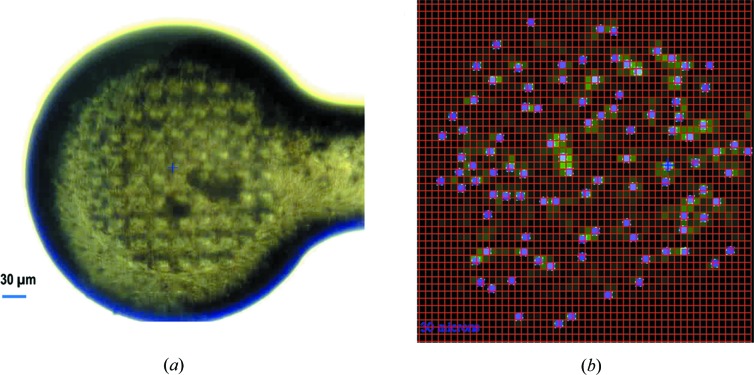
Microcrystal handling and diffraction data collection. (*a*) A representative image of a micro-sized wellmount loaded with microcrystals prior to a raster scanning. (*b*) Positions selected for data collection based on a raster-scanning heat map (green). Red meshes show the grid (55 µm) used for the scan. For this example, 101 positions (purple dots) were queued for data collection.

Microdiffraction data were collected at the FMX beamline at NSLS-II (Fuchs *et al.*, 2016 ▸). The beamline is equipped with an EIGER 16M detector which was calibrated to cover low-energy X-rays. We tuned the X-ray energy to 5 keV with a beam size of 5 × 9 µm. The estimated beam flux was about 6 × 10^10^ photons s^−1^. We collected all data sets in a way similar to that implemented in the MeshAndCollect method (Zander *et al.*, 2015 ▸). We aligned the wellmounts using a side view; and then rotated the mounts by 90° so that their surface was perpendicular to the X-rays [Fig. 1 ▸(*a*)]. We used raster scanning with a raster step size of 5 µm to find positions where diffraction data were collected. These positions, their diffraction intensities encoded as a green heat map, were visually checked for their diffraction quality and were used to guide the manual selection of positions to be queued for data collection [Fig. 1 ▸(*b*)]. From each position, we collected a partial rotation data set of 100 frames using rotation steps of 0.2° and an exposure time of 0.02 s per frame. The estimated cumulative dose is 5 MGy per crystal as calculated by the program *RADDOSE*-3*D* (Zeldin *et al.*, 2013*b* ▸). At a sample-to-detector distance of 140 mm, the corresponding Bragg spacing is about 3.0 Å at the detector edge. We collected a total of 1381 partial data sets from 18 wellmounts loaded with microcrystal slurries. On average, we collected 77 data sets on a single wellmount.

### 2.3. Data reduction and assembly   

**Figure 2:**
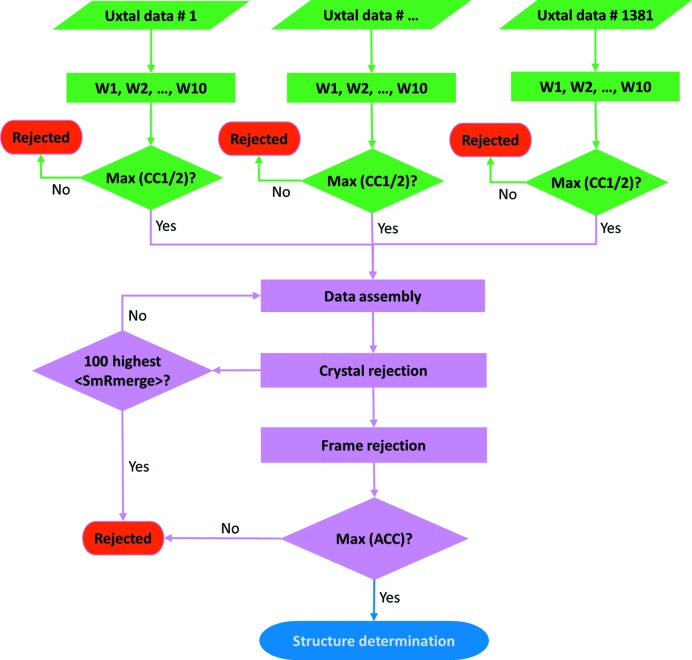
Schematic workflow of data assembly. Firstly, individual microcrystal data sets (100 frames each) were split and processed into ten accumulated wedges; and wedges with maximum CC_1/2_ were selected for data assembly. Secondly, iterative crystal rejection was performed by rejection of 100 microcrystals with the highest average SmRmerge, 〈SmRmerge〉. After each crystal rejection, frame rejection was performed to remove outliers based on defined SmRmerge cutoffs. Finally, the assembled data set with maximum ACC was used for structure determination.

Single-crystal data sets were indexed and integrated independently by using *DIALS* (Waterman *et al.*, 2016 ▸) and then scaled and merged to 2.6 Å spacings by using CCP4 programs *POINTLESS* and *AIMLESS* (Evans *et al.*, 2011 ▸; Evans & Murshudov, 2013 ▸). Our established method was used for outlier rejection and data assembly (Guo *et al.*, 2018 ▸). We processed single-crystal data sets as accumulated wedges of 10, 20, …, 100 frames, ending up with 10 wedges per crystal. Wedges with maximum CC_1/2_ calculated at 4 Å were chosen for subsequent data assembly. Smoothed frame *R*_merge_ (SmRmerge) as reported in *AIMLESS* (Evans & Murshudov, 2013 ▸) was used to score compatibility at both crystal and frame levels to guide further outlier rejections during data assembly. At the crystal level, average SmRmerge, 〈SmRmerge〉, was used to score each crystal and iterative crystal rejection was performed by rejecting 100 crystals with the highest 〈SmRmerge〉. The assembly of all 1381 single-crystal partial data sets was called ‘assembled data set 1381’. 〈SmRmerge〉 was calculated at each cycle to identify the most incompatible crystals for rejection in successive cycles. Starting from assembled data set 1381, we rejected 100 crystals with the highest 〈SmRmerge〉 and obtained assembled data set 1281, from which updated 〈SmRmerged〉 values were calculated for preparation of another cycle of rejection. By iteration, assembled data sets 1181, 1081, *etc*. were obtained until reaching the end with assembled data set 81. So at the crystal level, a total of 14 assembled data sets were obtained. At the frame level, we used SmRmerge per frame to reject frames based on radiation-induced decay. We rejected frames at different cutoffs as defined by frame_cutoff = [min(SmRmerge) × (1 + decay)], where min(SmRmerge) is the lowest SmRmerge within a single-crystal data set; and decay is a rejection ratio of none (effectively, decay = ∞), 500%, 200%, 150%, 100% or 50%. At each rejection ratio, frames with SmRmerge larger than frame_cutoff were excluded from assembly in *AIMLESS*. For example, a rejection ratio at 150% indicates that frames with a SmRmerge of 150% more than min(SmRmerge) are rejected from scaling and merging. We performed frame rejection at six ratios for each of the 14 assembled data sets (assembled data sets 1381, 1281, …, 81) and obtained a total of 84 assembled data sets with different extents of frame rejection. A schematic of our data assembly workflow is shown in Fig. 2 ▸. After crystal and frame rejection, the assembled data set 1181 with a frame-rejection ratio at 150% shows the highest anomalous correlation coefficient (ACC) (see details in results). The data-collection and refinement statistics for this assembled data set are shown in Table 1 ▸.

### 2.4. Structure determination   

Substructures were determined with *SHELXD* (Sheldrick, 2010 ▸). 5000 *SHELXD* trials were performed to search for nine anomalous scatterers with *E*_min_ cutoffs between 1.3 and 1.7 and with resolution cutoffs between 3.5 and 4.2 Å. The best substructure was used for calculating initial SAD phases in *PHASER* (Read & McCoy, 2011 ▸) or *SHARP* (Vonrhein *et al.*, 2007 ▸), followed by density modification with *DM* or *SOLOMON *(Cowtan & Zhang, 1999 ▸). Automatic model building was performed using *BUCCANEER* (Cowtan, 2006 ▸). Further iterative model building and refinement were performed in *COOT* and *PHENIX.REFINE*, respectively (Afonine *et al.*, 2012 ▸; Echols *et al.*, 2014 ▸). Bijvoet pairs were treated as two different reflections in all refinements, and the resultant Fourier coefficients were used for calculation of Bijvoet-difference Fourier maps. The stereochemistry of the refined structure was validated with *PROCHECK* (Laskowski *et al.*, 1993 ▸) and *MolProbity* (Chen *et al.*, 2010 ▸) for quality assurance. The refinement statistics for the assembled data set 1181 with 150% frame rejection are listed in Table 1 ▸.

## 3. Results   

### 3.1. Single-crystal data sets   

**Figure 3:**
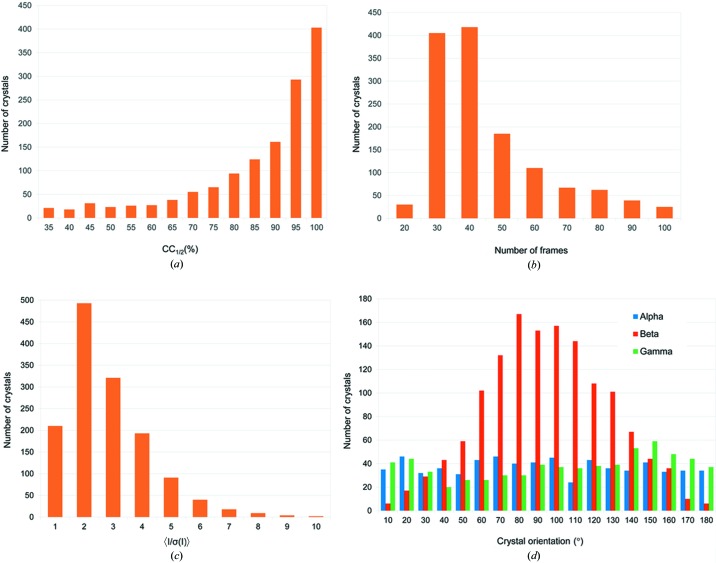
Data analysis of single-crystal data sets. (*a*) CC_1/2_ distribution of single crystals. (*b*) Distribution of the number of frames selected from single crystals. (*c*) 〈*I*/σ(*I*)〉 distribution of single crystals. (*d*) Distribution of crystal orientations shown as three Euler angles α, β, and γ.

The required X-ray dose for a given signal-to-noise ratio is inversely proportional to the volume of microcrystals (Holton & Frankel, 2010 ▸). Therefore, to obtain diffraction spots to the required Bragg spacings, only an incomplete partial data set could be collected from each microcrystal before its being killed by radiation damage. We used CC_1/2_ at 4.0 Å to evaluate single-crystal data quality and did initial rejection to remove frames which caused the decrease of CC_1/2_ if they were combined. By this initial frame rejection at a lower resolution, we made a compromise of having more frames to be selected for downstream assembly, while rejecting frames with too much radiation damage which would make the scaling process unstable if they were not rejected. Fig. 3 ▸(*a*) shows the distribution of microcrystals with respect to CC_1/2_ after the initial frame rejection. With the maximum CC_1/2_ as criteria, we can select 20 to 100 frames per crystal, indicating that single crystals respond differently to radiation damage, with 30 to 40 frames selected for most single crystals [Fig. 3 ▸(*b*)]. After the initial frame rejection, we selected a total of 51 570 frames from 1 381 crystals for subsequent data assembly. The diffraction power of these microcrystals is weak. Necessarily the average *I/*σ(average *I*), 〈*I*/σ(*I*)〉, of these single-crystal data sets tends to be low. For the 1381 single-crystal data sets, most display an 〈*I*/σ(*I*)〉 of about 2 [Fig. 3 ▸(*c*)].

Crystal morphology and distribution on the support could impact the reciprocal-space coverage and data assembly. We used patterned microwells to distribute the orientations of microcrystals on the support. To check the orientation distribution of the microcrystals, we indexed single-crystal data sets, converted their orientation matrices to three Euler angles and plotted the angular distribution in Fig. 3 ▸(*d*). Euler angles α and γ have a roughly uniform distribution between 0 and 180° but there is a skewed distribution for β peaked at ∼80°. Considering that the lattice of thaumatin crystals is tetragonal, the skewed distribution of β can be well accommodated by symmetry-related measurements.

### 3.2. Assembled data sets   

**Figure 4:**
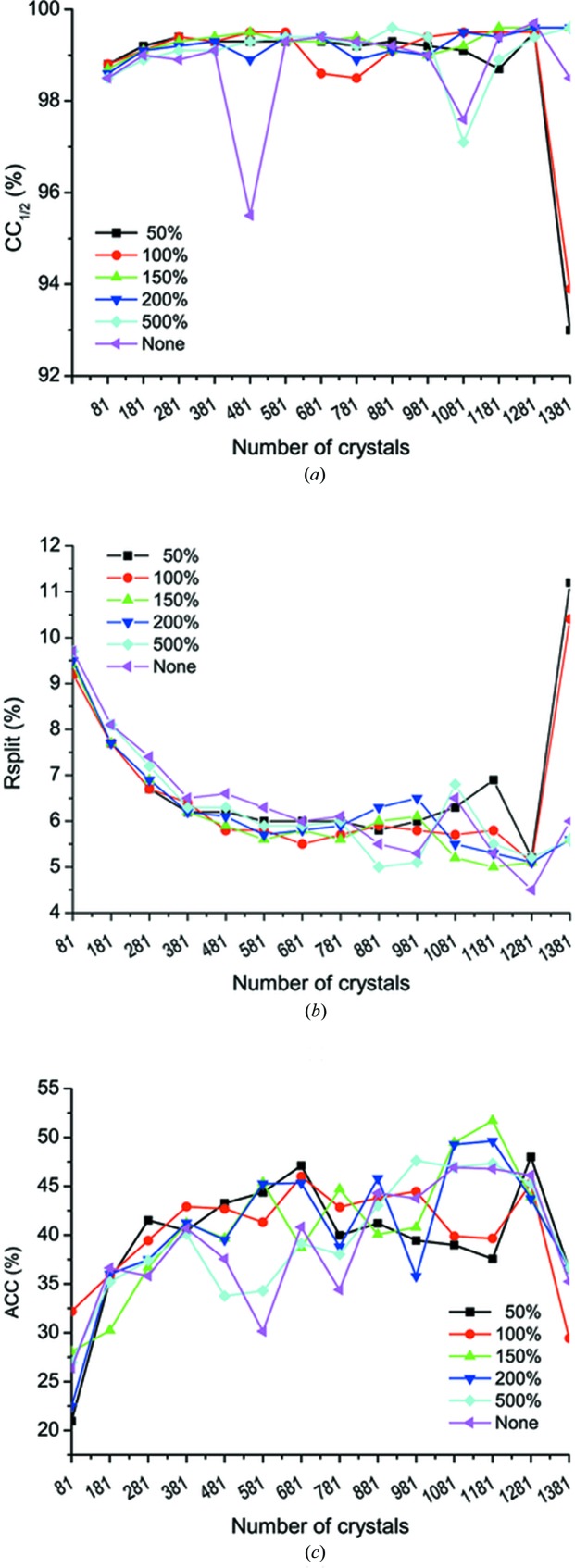
Data analysis of assembled data sets. (*a*) CC_1/2_, (*b*) *R*_split_ and (*c*) ACC. Within each plot, the curves are corresponding to a different extent of frame rejection after each cycle of crystal rejection. Frame rejection is shown at six different ratios with 50% being the most stringent rejection and ‘None’ being no frame rejection. ACC values were calculated with high-resolution data truncated to 4.0 Å.

We combined the 1381 partial single-crystal data sets and rejected outlier crystals and frames progressively. Because of data incompleteness in each single-crystal data set and missing cross-crystal reflections for a reliable correlation analysis, we used SmRmerge which indicates the overall compatibility of each frame to the merged data set. By using 〈SmRmerge〉, we sorted the 1381 single-crystal data sets and iteratively rejected 100 crystals of the highest 〈SmRmerge〉. In two iterations, such crystal rejection removed the 200 most statistically incompatible crystals which are detrimental to CC_1/2_, *R*_split_ and ACC (assembled data sets 1281 and 1381) (Fig. 4 ▸). For each of these 14 assembled data sets, we did frame rejections at six ratios based on SmRmerge. By combination of the two rejection strategies, we found that significant anomalous signals can be extracted from 1 081 or 1 181 crystals with frame-rejection ratios of 150% or 200% [Fig. 4 ▸(*c*)]. Assembled data set 1181 with a frame-rejection ratio at 150% gave the highest ACC of 51.7%. This data set was then used in structure determination. We also found that stringent frame rejections at 50% and 100% reduced anomalous signals. Because of variation among microcrystals, we suggest conducting frame rejection at different ratios and selecting the one with the highest ACC for structure analysis.

### 3.3. SAD phasing and anomalous signals   

**Figure 5:**
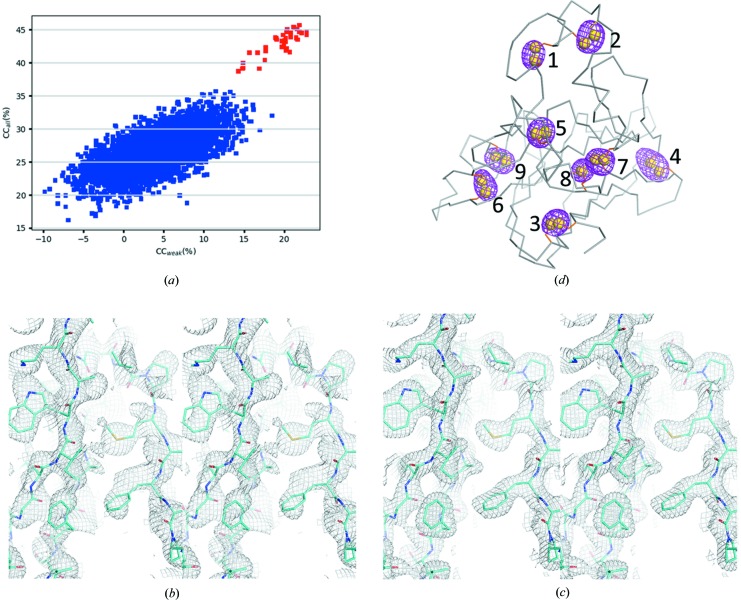
Structure determination and phasing. (*a*) CC_all_/CC_weak_ of 5000 *SHELXD* trials, (*b*) experimental electron density after density modification, (*c*) refined electron-density map and (*d*) Bijvoet-difference Fourier peaks. Peaks for anomalous scatterers (sulfur) are shown as magenta isomeshes contoured at 3σ. The overall structure of thaumatin is shown as ribbons. The numbers and yellow spheres indicate, respectively, the positions and atoms of anomalous scatters in the structure: (1) Cys149–Cys158, (2) Cys159–Cys164, (3) Cys9–Cys204, (4) Cys121–Cys193, (5) Cys134–Cys145, (6) Cys56–Cys66, (7) Cys126–Cys177, (8) Met122, (9) Cys71–Cys77.

Thaumatin contains eight di­sulfide bonds and one me­thio­nine residue for a total of 17 sulfur atoms. With a resolution cutoff at 4.0 Å, di­sulfide bonds (2.02 Å) are not resolvable and we thus searched for nine sites in *SHELXD*. With an *E*_min_ cutoff at 1.4 and 5000 *SHELXD* trials, we obtained 41 substructure solutions with the highest CC_all_ and CC_weak_ of 45.7% and 22.8%, respectively [Fig. 5 ▸(*a*)]. Substructures were used for SAD phasing using *SHARP* (Vonrhein *et al.*, 2007 ▸). After density modification, electron-density maps at 2.6 Å resolution were of sufficient quality for model building in *COOT* [Fig. 5 ▸(*b*)]. The program *BUCCANEER* (Cowtan, 2006 ▸) was able to build 178 out of 202 residues automatically. The refined structure has an *R*/*R*_free_ of 18.6/21.6%, indicating the data quality of this assembled data. In comparison to the experimental electron density, Fig. 5 ▸(*c*) shows the electron density of the refined map.

To evaluate the strength of anomalous signals, we carried out f" refinement (Liu *et al.*, 2013 ▸) for the eight di­sulfide bonds and the one me­thio­nine in *PHENIX.REFINE* by using Bijvoet pairs. The highest f" value is 1.34 e for Cys149–Cys158, and the lowest f" is 0.96 e for Cys56–Cys66, with an average of 1.14 e for all sulfur atoms. At 5 keV, the theoretical f" for sulfur is 1.31 e. Therefore, our low-energy experiment has preserved the majority of the anomalous diffraction signals. Consequently, all nine Bijvoet-difference Fourier peaks can be seen clearly beyond 3.0σ with the highest peak at 10σ for Cys121–Cys193 [Fig. 5 ▸(*d*)]. The success in structure determination and the strengths of the anomalous signals demonstrate that native SAD is possible at a synchrotron beamline from about 1 200 crystals of less than 10 µm in size.

### 3.4. Frame rejection and radiation damage   

**Figure 6:**
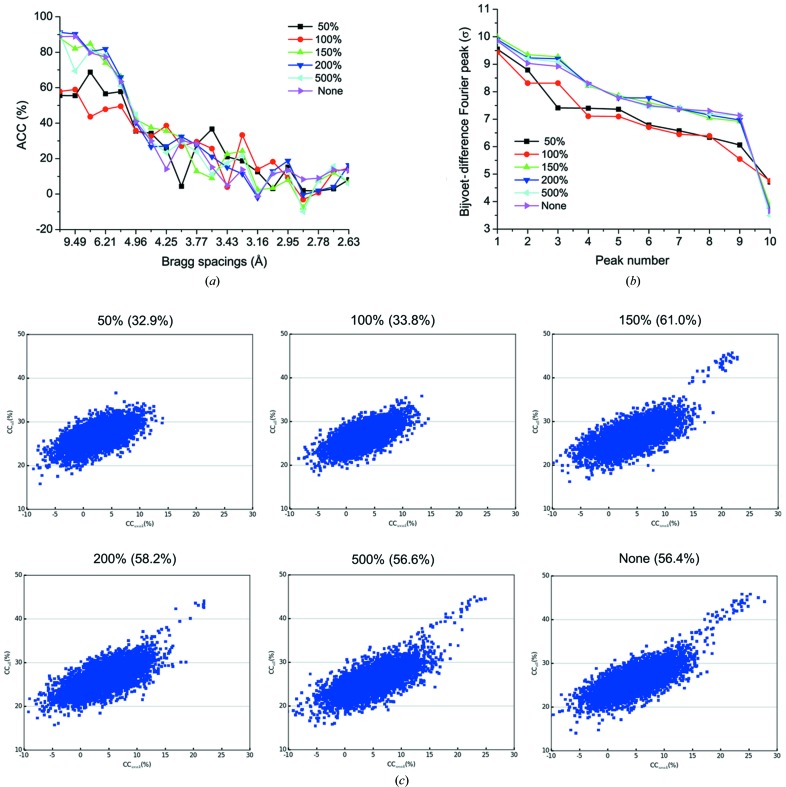
Analysis of the assembled data set 1181. (*a*) Plot of ACC(%) with respect to Bragg spacings. (*b*) Plot of the ten highest Bijvoet-difference Fourier peaks. The first nine peaks (peaks 1–9) are from nine anomalous scatterers and the tenth peak is from noise. (*c*) Plot of CC_all_/CC_weak_ from *SHELXD* substructure determination for data set 1181 with different frame-rejection ratios. For each data set, 5000 *SHELXD* trials were performed with *E*_min_ = 1.4 and resolution cutoff = 4.0. MR-SAD with the known structure was used for SAD phasing and the mapCC after density modification are shown in parentheses.

We explored the impact of frame-rejection ratios on assembled data set 1181. We found that ACCs and Bijvoet-difference Fourier peaks are dramatically affected [Figs. 6 ▸(*a*) and 6(*b*)]. With a rejection ratio of 100% or 50%, *i.e.* with fewer damaged data being included in the assembled data set, ACC decreased substantially for low-resolution shells (*d*_min_ > 5 Å). Bijvoet-difference Fourier peaks and the gap between peak 9 (the lowest anomalous peak) and peak 10 (the highest background peak) also decreased perhaps because of deteriorated low-resolution anomalous signals. With a rejection ratio of 150% or higher, low-resolution anomalous signals were preserved with neither much variation of Bijvoet-difference Fourier peaks nor the ACC values. This observation suggests that radiation-damaged frames still contributed to the enhancement of low-resolution anomalous signals and should be preserved during data processing. We attribute such enhancement to the enhanced multiplicity, which improved the signal-to-noise ratio through averaging. In addition, radiation damage, including non-isomorphism that it may induce, affects high-angle data first. Although our dose of 5 MGy per crystal is well below the 30 MGy Garman limit (Garman & Weik, 2017 ▸) and 20 MGy Henderson limit (Henderson, 1990 ▸), it is widely recognized that a more stringent level is required to preserve weak anomalous signals.

To further investigate the impact of frame rejection on native-SAD structure determination, we ran substructure searches by using *SHELXD* for assembled data set 1181 with different frame-rejection ratios. We found that preserving low-resolution anomalous signals is correlated with success in substructure determination [Fig. 6 ▸(*c*)]. As may be seen, with rejection ratios of 50% and 100% there are no substructure solutions. As a contrast, assembled data sets with frame-rejection ratios of 150% or more, including no rejection, yield substructure solutions. This result emphasizes that proper frame rejection is important for substructure determination from microcrystals.

To evaluate the effect of frame rejection on structure solvability, we used molecular-replacement SAD (MR-SAD) (Read & McCoy, 2011 ▸) to calculate SAD phases starting with the refined structure. After density modification of SAD phases, we calculated the map correlation coefficient (mapCC) between the SAD and the model map. We found that a rejection ratio of 150% or higher gave a mapCC of 56% or more. However, assembled data sets with rejection ratios of 50% and 100% have mapCC of about 33%, indicating much weaker anomalous signals arising from too stringent frame rejection, even though the radiation damage is necessarily less in these two data sets. Therefore, for microcrystal native-SAD phasing at a synchrotron with rotation data collection, proper frame rejection is necessary. It is vital to use a less stringent frame-rejection ratio in order to enhance low-resolution anomalous signals for substructure determination and phasing.

## 4. Discussion   

### 4.1. Microdiffraction data collection   

With microcrystals less than 10 µm in size, we have demonstrated the capability of synchrotron native-SAD phasing at a lower energy of 5 keV. Our experiment used ∼1 400 microcrystals from which 1 181 crystals were used for an optimal data assembly. To collect thousands of data sets, an efficient data-collection strategy is necessary which requires high-density crystal mounts with fast microcrystal identification and collection. To increase the crystal density on wellmounts, we concentrated microcrystals, removed as much supernatant as possible without touching the crystal slurry, and obtained on average 77 data sets per wellmount. The highest yield we achieved had 101 data sets on the wellmount.

We used a beam size of 5 × 9 µm for data collection. To prevent X-ray damage to nearby regions, we selected diffraction positions separated by at least one mesh grid (5 µm in the horizontal) [Fig. 1 ▸(*c*)]. This microdiffraction data-collection strategy could be automated as implemented at the European Synchrotron Radiation Facility (Zander *et al.*, 2015 ▸) and the Swiss Light Source (Basu *et al.*, 2019 ▸). Preliminary analysis of raster-scanned crystals may provide useful information to assist designing data-collection strategies, for example by preventing collecting data from overlapped crystals (Melnikov *et al.*, 2018 ▸). Steps of crystal identification and rotation data collection could be combined as originally proposed and tested (Gati *et al.*, 2014 ▸). This mode of data collection reduces unnecessary radiation damage but with a compromise of having a lot of empty frames that have to be identified and rejected prior to standard diffraction data analysis (Gao *et al.*, 2018 ▸).

### 4.2. Radiation damage   

In order to extract weak anomalous signals for microcrystal native-SAD phasing, radiation damage is one major challenge. To squeeze out the most data from a single crystal and subsequently to extract weak anomalous signals, we took three approaches. The first approach is to overexpose each microcrystal by collecting more frames per sample. As shown in Fig. 3 ▸(*b*), although most microcrystals only allowed for optimal data with wedges containing 30–40 frames, we collected 100 frames from which an optimal wedge could be selected. The second approach is to process single-crystal data sets progressively into successive data wedges. This assures to have as many frames as possible to be included by using maximum CC_1/2_ at a lowered resolution (4 Å for thaumatin) [Fig. 3 ▸(*b*)]. The third strategy is to fine-tune the rejection of radiation-damaged frames after each cycle of crystal rejection (Fig. 4 ▸). We found that combining these strategies can effectively treat radiation damage while extracting the most anomalous diffraction signal from each single crystal.

Through our frame-rejection analysis, we found that including more data, even though damaged, is beneficial to enhance low-resolution anomalous signals for substructure determination. For example, on *SHELXD* substructure determination, the highest CC_all_/CC_weak_ (45.8%/27.8%) is from the assembled data set 1181 with no frame rejection. We did substructure searches using reflections out to 4.0 Å spacings; while the SAD phases were calculated and density modifications were performed with all reflections out to 2.6 Å. In terms of mapCC, the best phases (61%) are from 150% frame rejection.

### 4.3. Low-energy X-rays   

Low-energy X-rays are very attractive for native-SAD phasing because of the increased f" for sulfur and phospho­rus in native biomacromolecules (Hendrickson, 2014 ▸; Liu & Hendrickson, 2015 ▸). In addition, overall diffraction signals at a lower energy are much stronger, proportional to the cube of the corresponding wavelength (Holton & Frankel, 2010 ▸). However, absorption of low-energy X-rays, including anomalous signals, also increases with decreased energy. The absorption may come from the crystal itself, the crystal support (mount), the path between crystal and detector, and the detector sensor material. In our 5 keV experiment, we used microcrystals of a few micrometres. The sample absorption was calculated to be less than 2% and could thus be ignored. To reduce absorption from the support, we used polyimide wellmounts of about 3 µm thick which can transmit 99% of X-rays at 5 keV. Therefore, our polyimide mounts are well suited for low-energy native-SAD experiments. At 5.4 keV, the specified detective quantum efficiency for the EIGER 16M is 94%, which is close to the 5 keV that we used. By using the setting calibrated at 5 keV, the sensor absorption should not impact our low-energy experiment. It is noted that we did not use a helium environment to reduce air absorption. Our sample-to-detector distance is 140 mm, which may contribute to air absorption of 5 keV X-rays by 46.3%. Nevertheless, structure determination by microcrystal native-SAD phasing proved possible at 5 keV even without a helium environment. Instrumentation of a helium or vacuum environment would certainly facilitate native-SAD phasing at low energy as implemented at the Diamond Light Source (Wagner *et al.*, 2016 ▸) and the Photon Factory (Liebschner *et al.*, 2016 ▸).

Measurement at an X-ray wavelength of 2.1 Å (*E* = 5.9 keV) has been proposed as optimal for native-SAD phasing, although the possible variation of this optimum with crystal size was not explored (Mueller-Dieckmann *et al.*, 2005 ▸). By using a multi-crystal strategy, we also showed that the anomalous signals recorded from data collected at 6 keV are superior to those collected at 7 keV (Liu *et al.*, 2014 ▸). In consideration of the crystal-size-dependent absorption of low-energy X-rays, using an energy below 6 keV has been proposed for microcrystals (Liu *et al.*, 2014 ▸; Liebschner *et al.*, 2016 ▸; Wagner *et al.*, 2016 ▸). Even with relatively large crystals (50 µm), it has been shown that a longer wavelength of 2.7 Å (*E* = 4.6 keV) is superior to a shorter wavelength of 1.9 Å (*E* = 6.5 keV) for native-SAD phasing (Liebschner *et al.*, 2016 ▸). In our microcrystal native-SAD experiments at a low energy, we used an energy at 5 keV, the low-energy limit of the photon delivery system of the FMX beamline. Similar to what have been done for larger crystals, it may be interesting to explore the native-SAD phasing efficacy of microcrystals with respect to X-ray energies between 7 and 4 keV, although such a study is likely to need additional instrumentation not currently available at FMX.

### 4.4. Multiple crystals and outlier rejection   

The use of multiple crystals provides an efficient way of obtaining the necessary multiplicity and data accuracy for enhancing anomalous signals for *de novo* structure determination (Liu *et al.*, 2011 ▸; Liu & Hendrickson, 2017 ▸). One major problem arising from using multiple crystals is the variation among crystals which compromises the data merging if not treated properly. We and others have proposed to use unit-cell variation, diffraction-intensity dissimilarity and relative contribution of ACCs for rejection of outlier crystals (Giordano *et al.*, 2012 ▸; Liu *et al.*, 2012 ▸; Foadi *et al.*, 2013 ▸). For microcrystals, only a partial data set is achievable per crystal; thus multiple crystals must be used and outlier rejection needs to be performed based on unit-cell variation or correlation to a merged or reference data set (Axford *et al.*, 2015 ▸; Guo *et al.*, 2018 ▸; Yamashita *et al.*, 2018 ▸; Basu *et al.*, 2019 ▸). The use of genetic algorithm classification and outlier crystal rejection provide an alternative way for finding compatible partial data sets (Zander *et al.*, 2016 ▸). In our experience, the unit-cell dimensions of thaumatin microcrystal do not vary much (Guo *et al.*, 2018 ▸). Consequently, in our 5 keV experiment, we did not use unit-cell variation for initial rejection of non-isomorphic data. Instead, we combined crystal and frame-rejection steps with the data-assembly process, thus simplifying the data-analysis workflow. By using maximum CC_1/2_ at 4 Å for initial frame rejection, we also did not use the reference data set, further simplifying and speeding up data assembly. Selection of resolution cutoff at 4 Å so far is empirical. Using a different maximum CC_1/2_ cutoff would affect the total number of frames being included for assembling and subsequent crystal and frame rejection. Based on our experience, one could also use several resolution cutoffs to optimize the frame selection in single-crystal data sets.

## 5. Concluding remarks   

Native-SAD phasing from microcrystals of less than 10 µm in size is challenging and was not yet routinely demonstrated at a synchrotron source. Here we show that native-SAD phasing from such microcrystals is feasible at a synchrotron beamline by using as few as 1 200 crystals with a multiplicity of 323. The use of low-energy X-rays at 5 keV, low-absorbance polyimide wellmounts and iterative outlier rejections make microcrystal native-SAD phasing promising for real-life applications on challenging samples such as microcrystals of membrane proteins and complexes. Native-SAD phasing with larger crystals may also benefit from such low-energy experiment and analysis.

## Supplementary Material

PDB reference: thaumatin, 6o8a

## Figures and Tables

**Table 1:** Data-collection and refinement statistics for the merged data set 1181 with 150% frame rejection Values in parentheses are for the highest resolution range.

Data collection	
Beamline	FMX (NSLS-II)
Wavelength (Å)	2.48
Space group	*P*4_1_2_1_2
Cell dimensions *a*, *c* (Å)	57.70, 150.45
Solvent content (%)	53.0
Bragg spacings (Å)	40–2.63 (2.70–2.63)
Total reflections	2721845
Unique reflections†	8435 (14853)
Completeness (%)	100.0 (100.0)
〈*I*/σ(*I*)〉	22.4 (2.1)
*R*_split_	0.05 (0.583)
Multiplicity	322.7 (46.8)
CC_1/2_	0.996 (0.684)
Refinement	
Resolution (Å)	2.6
No. reflections	8379
*R*_work_/*R*_free_	0.186/0.216
No. atoms	1654
Wilson *B* factor (Å^2^)	24.1
Average *B* factor (Å^2^)	23.7
R.m.s deviations	
Bond length (Å)	0.002
Bond angle (°)	0.435
PDB code	6o8a

† In parentheses is the number of unique reflections with Bijvoet pairs separated.
